# Phosphite binding by the HtxB periplasmic binding protein depends on the protonation state of the ligand

**DOI:** 10.1038/s41598-019-46557-2

**Published:** 2019-07-15

**Authors:** Nathan B. P. Adams, Angus J. Robertson, C. Neil Hunter, Andrew Hitchcock, Claudine Bisson

**Affiliations:** 1 Department of Molecular Biology and Biotechnology, The University of Sheffield, Firth Court, Western Bank, Sheffield, S10 2TN UK; 2 Department of Biological Sciences, ISMB, Birkbeck College, University of London, Malet Street, London, WC1E 7HX UK

**Keywords:** X-ray crystallography, NMR spectroscopy, Environmental microbiology, Proteins, Molecular engineering

## Abstract

Phosphorus acquisition is critical for life. In low phosphate conditions, some species of bacteria have evolved mechanisms to import reduced phosphorus compounds, such as phosphite and hypophosphite, as alternative phosphorus sources. Uptake is facilitated by high-affinity periplasmic binding proteins (PBPs) that bind cargo in the periplasm and shuttle it to an ATP-binding cassette (ABC)-transporter in the bacterial inner membrane. PtxB and HtxB are the PBPs responsible for binding phosphite and hypophosphite, respectively. They recognize the P-H bond of phosphite/hypophosphite via a conserved P-H...*π* interaction, which confers nanomolar dissociation constants for their respective ligands. PtxB also has a low-level binding affinity for phosphate and hypophosphite, whilst HtxB can facilitate phosphite uptake *in vivo*. However, HtxB does not bind phosphate, thus the HtxBCDE transporter has recently been successfully exploited for biocontainment of genetically modified organisms by phosphite-dependent growth. Here we use a combination of X-ray crystallography, NMR and Microscale Thermophoresis to show that phosphite binding to HtxB depends on the protonation state of the ligand, suggesting that pH may effect the efficiency of phosphite uptake by HtxB in biotechnology applications.

## Introduction

Phosphorus is a core building block of life. It is required for storage and exchange of genetic information, energy generation, cellular metabolism and membrane integrity^1^, thus phosphorus acquisition is a critical process for all living organisms. In natural environments, phosphorus is predominantly found as inorganic phosphate (PO_4_^3−^), which is the only form of the element that cells can utilise directly for biological processes^1–3^. However, phosphate availability varies significantly in different ecosystems and despite the presence of high-affinity phosphate uptake mechanisms in many microorganisms, a lack of phosphate limits microbial growth. To overcome this limitation, some bacteria can import and metabolise reduced phosphorus compounds, such as phosphite (HPO_3_^2−^), hypophosphite (H_2_PO_2_^−^) and organophosphonates^2,4,5^. The Gram-negative soil bacterium *Pseudomonas stutzeri* WM88 is an example of an organism that can utilise these compounds as growth-supporting phosphorus sources^6–9^. Following uptake, phosphite is oxidised to phosphate by an NAD:phosphite oxidoreductase/phosphite dehydrogenase (PtxD)^6,10,11^. Hypophosphite is oxidised to phosphite by hypophosphite dioxygenase (HtxA), which is subsequently converted to phosphate by PtxD. Conversely, phosphonates are catabolised by the carbon-phosphorus (C-P) lyase machinery, producing 5-phosphoribosyl-*α*-1-diphosphate (PRPP) in an ATP-dependent fashion^12^.

As phosphite is water-soluble, kinetically stable, non-toxic, and inexpensive, it is increasingly utilised as a phosphorus source in biotechnological and agricultural applications. Engineered phosphite-dependent growth has been reported in the model heterotrophic organism *Esherichia coli*^13^ and in cyanobacteria^14,15^, algae^16,17^, plants (for reviews see^18,19^) and yeast^20,21^. Although recognised as a component of the global biological phosphorus redox cycle^22^, phosphite is scarce in natural environments, making it an ideal phosphorus source for processes that need to avoid contamination (e.g. open ponds, large-scale fermentation) and/or for biocontainment of genetically modified organisms^13,21^.

Microorganisms use ATP-binding cassette (ABC)-transporters for the active uptake of solutes from the environment. *P. stutzeri* WM88 has PtxABC, HtxBCDE and PhnCDE ABC-transporters for the acquisition of phosphite, hypophosphite and organophosphonates, respectively^2,10,14,23^. These transport systems rely on a high-affinity periplasmic binding protein (PBP) to impart ligand specificity. We have previously reported binding affinities of phosphorus ligands to the purified recombinant PBPs of the *P. stutzeri* PtxABC (PBP = PtxB) and HtxBCDE (PBP = HtxB) transporters, and determined X-ray crystal structures of the ligand-bound proteins^24^. The structures of PtxB with phosphite and HtxB with hypophosphite revealed that a P-H…*π* interaction between the ligand and the protein confers substrate specificity in both PBPs, representing a conserved mechanism for selection of a ligand with a P-H bond over phosphate or organophosphonates. We used Microscale Thermophoresis (MST) to record nanomolar dissociation constants (*K*_d_) of PtxB for phosphite and HtxB for hypophosphite, with other, bulkier ligands having ≈2–3 orders of magnitude lower affinity. Although not physiological relevant, lower affinity phosphate binding by PtxB allows phosphate uptake by PtxABC *in vivo* when it is heterologously produced in *E. coli*, making this transporter inappropriate for biocontainment stragtegies^13^.

**Figure 1:**
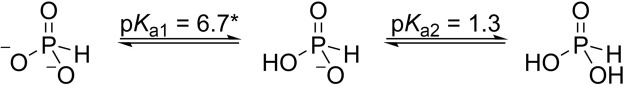
Chemical structures and p*K*_a_s of phosphite. *In this study, p*K*_a1_ was determined to be 6.3 by NMR.

We found that HtxB does not bind phosphate and we could not detect binding of phosphite up to ligand concentrations of 10 mM via MST at neutral pH^24^. A comparison of the HtxB and PtxB structures showed that sequence differences within the binding pocket essentially eliminate an oxygen binding site in HtxB, providing specificity for a ligand with two oxygen atoms (hypophosphite), over one with three (phosphite). Consistent with this, the volume of the binding pocket in HtxB is approximately 35% smaller than that of PtxB, favouring a smaller ligand^24^. However, experiments by others have shown that the HtxBCDE transporter allows uptake of phosphite as well as hypophosphite in engineered microorganisms *in vivo*^13,15^. Coupled with genetic modification to remove native phosphate uptake systems, this makes HtxBCDE an ideal transporter to use in combination with PtxD for conditional growth on phosphite, enabling phosphite-dependent biocontainment. Such system have recently been engineered into both *E. coli*^13^ and a model cyanobacterium, *Synechococcus elongatus* PCC 7942^15^. These studies show that the HtxBCDE transporter allows phosphite uptake *in vivo* at media ligand concentrations of 0.2–1 mM, concentrations at which we should have detected binding in our *in vitro* assays with recombinant HtxB. Phosphite binding to HtxB therefore warranted further investigation; here we present new characterization of ligand binding to HtxB, showing how pH modulates substrate specificity due to a change in the protonation state of the ligand (Fig. 1).

## HtxB can form a Complex with Mono-Anionic Phosphite at Low pH

In a previous study we determined the structure of *P. stutzeri* HtxB in complex with hypophosphite (PDB:5ME4), but under the crystallisation conditions used, we were unable to obtain the complex with phosphite^24^. We have now obtained a 1.25 Å structure of the HtxB/phosphite complex (PDB:6EMN) (see Table 1 for data collection and refinement statistics), which was crystallised at pH 5, showing how HtxB accommodates phosphite in its binding pocket.

**Table 1:** ^a^R_merge_ = Σ_hkl_ Σ_I_|I_i_ − I_m_|/Σ_hkl_ Σ_i_ I_i_.

Data Collection	HtxB + phosphite (PDB:6EMN)	D206A HtxB + hypophosphite (PDB:6GHT)	D206N HtxB (PDB:6GHQ)
Beamline	DLS, i04-1	DLS, i04	DLS, i03
Wavelength (Å)	0.92819	0.97951	0.9718
Resolution (Å)	50.62–1.25	50.64–1.12	39.49–1.53
	(1.27–1.25)	(1.139–1.12)	(1.56–1.53)
Space group	P2_1_2_1_2_1_	P2_1_2_1_2_1_	P2_1_2_1_2
Unit cell dimensions (a, b, c, *α*, *β*, *γ*)	40.28, 55.2, 126.33, 90, 90, 90	40.08, 55.24, 125.9, 90, 90, 90	70.82, 118.47, 35.44, 90, 90, 90
Total reflections^c^	463423 (17894)	885584 (23864)	319442 (45946)
Unique reflections^c^	78831 (3871)	108281 (5252)	45946 (2520)
Multiplicity^c^	5.9 (4.6)	8.2 (4.5)	6.7 (6.1)
Completeness^c^ (%)	100 (99.9)	99.9 (98.7)	99.9 (99.6)
Mean I/*σ*^c^ (I)	9.3 (1.0)	10.7 (0.4)	19.1 (1.4)
CC half^c^	0.998 (0.403)	0.999 (0.322)	0.999 (0.602)
R_merge_^a,c^	0.094 (1.585)	0.071 (2.78)	0.037 (1.093)
R_pim_^b,c^	0.046 (0.890)	0.027 (1.59)	0.017 (0.517)
**Refinement**			
R_factor_	0.15	0.16	0.19
R_free_	0.17	0.19	0.21
RMSD bonds (Å)	0.0152	0.0114	0.0099
RMSD angles (°)	1.53	1.58	1.46
**No. of non-H atoms**			
Protein	2028	2080	2087
Ligands	13	11	27
Water	238	243	225
Protein residues	255 (Chain A; 7–262)	260 (Chain A; 2–262)	266 (Chain A; 2–268)
**Average B factors**			
Main chain	15.7	16.7	28.4
Side chains	22.4	22.3	36.2
Ligands	18.4	20.1	40.0
Water	33.8	33.4	43.8
**Ramachandran**			
favored/allowed (%)	97.7/2.3	97.7/2.3	98.1/1.9
Molprobity score	1.02 (99^th^ percentile; N = 2054, 1.25 ± 0.25 Å)	1.28 (89^th^ percentile; N = 958, 1.12 ± 0.25 Å)	1.02 (99^th^ percentile; N = 4917, 1.53 ± 0.25 Å)

^b^R_pim_ = Σ_hkl_ $\surd 1$/n − 1 Σ_i=1_|I_i_ − I_m_|/Σ_hkl_ Σ_i_ I_i_, where I_i_ and I_m_ are the observed intensity and mean intensity of related reflections, respectively. ^c^Values in parenthesis are for data in the high-resolution shell.

**Figure 2:**
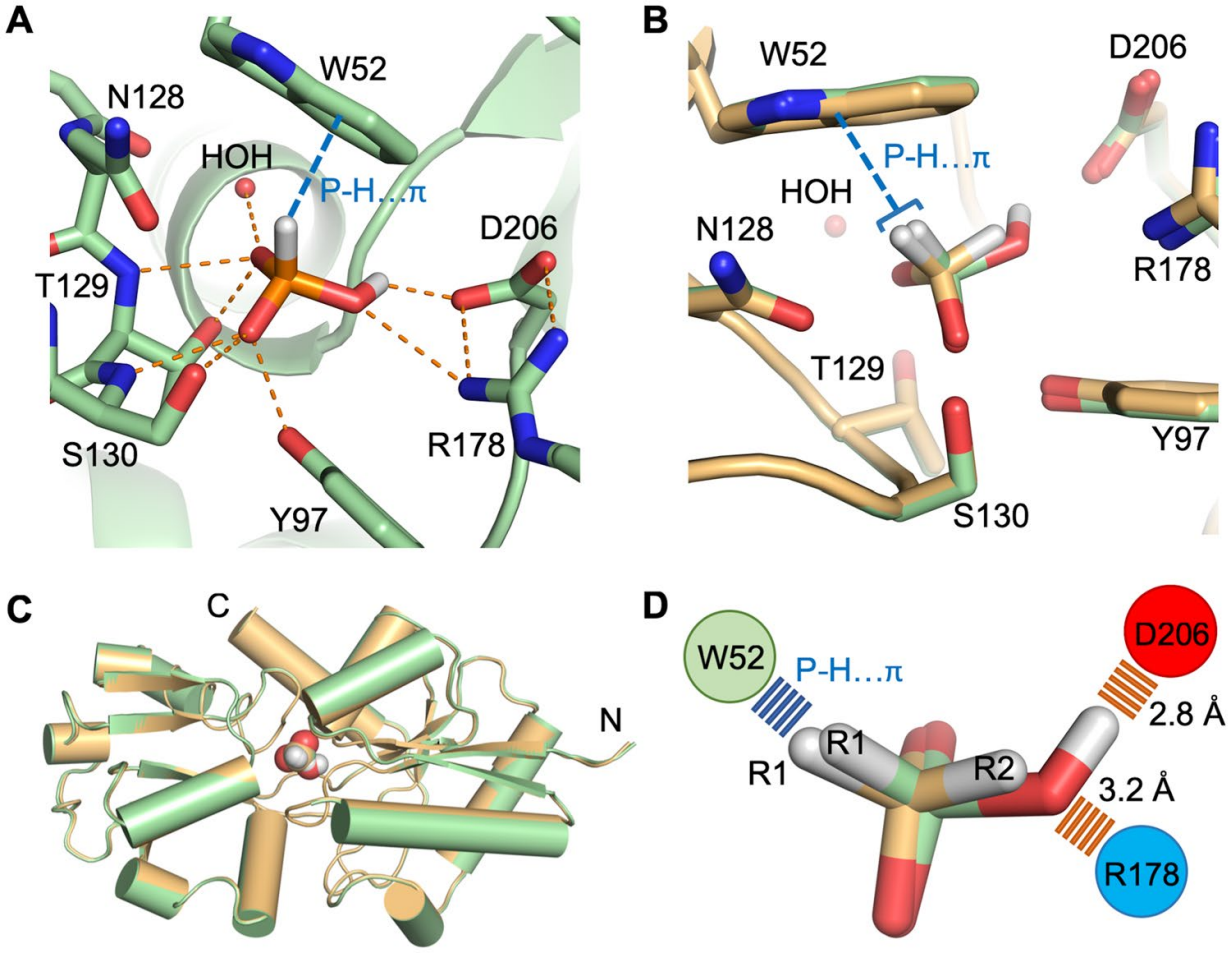
HtxB binds phosphite at pH 5 via hydrogen bonds to D206 and R178. (**A**) The hydrogen bonding network surrounding phosphite in the HtxB binding pocket. The phosphite acts as a hydrogen bond donor via the protonated oxygen, forming an interaction with the presumably deprotonated sidechain of D206 (2.8 Å). It also acts as a hydrogen bond acceptor to R178, forming a rather long hydrogen bonding interaction (3.2 Å). (**B**) Superposition of the HtxB complex with phosphite (green) and hypophosphite (orange) showing the conserved position of residues surrounding the binding site and the conserved P-H…*π* bond with W52. In (**A**,**B**) the protein is drawn as a cartoon with the ligands and selected side chains drawn as sticks. The single water in the binding pocket is drawn as a small red sphere. The P-H…*π* interaction is drawn as a blue dashed line and hydrogen bonds are drawn as orange dashed lines. (**C**) The same superposition shows that the fold and conformation of the protein is practically identical in complex with phosphite (green) or hypophosphite (orange). The protein backbone is represented as a cartoon and the ligands are drawn as spheres. (**D**) A schematic showing that R1 is positionally conserved in both phosphite (green) and hypophosphite (orange), with the hydrogen at the R2 position in hypophosphite being replaced by the oxygen atom of the hydroxyl that hydrogen bonds to D206 and R178 in the HtxB-phosphite complex. Note that the hydrogen position on the R2 oxygen of the phosphite is assumed based in the hydrogen bonding network, it cannot be observed in the electron density map. The ligands are shown as sticks and the relative position and interactions with W52, D206 and R178 are indicated schematically with circles and dashed lines, respectively.

HtxB is a typical type-II PBP, with a binding pocket positioned in a deep groove between two domains separated by a hinge region. In the HtxB complex with phosphite, the phosphorus centre of the ligand and the four tetrahedral atoms surrounding it (3 oxygen and 1 hydrogen) occupy approximately the same positions as the equivalent atoms in the hypophosphite complex (2 oxygen and 2 hydrogen). The two common oxygen atom positions between the phosphite and hypophosphite make equivalent interactions with residues in the binding pocket, namely the sidechain and mainchain amide of T129, S130, the sidechain hydroxyl of Y97 and the structural water molecule (HOH) (Fig. 2A–C). The walls of the binding pocket are formed by packing interactions between P71, N128, F158, M176, R178 and D206, with the latter two resides forming a buried salt bridge. The pocket is capped by a tryptophan sidechain (W52) that makes a P-H…*π* interaction with the R1 hydrogen atom of the phosphite (distance of 2.7 Å and angle of approximately 140°), forming a similar interaction to that present in the hypophosphite complex. The R2 hydrogen in hypophosphite is not involved in any hydrogen bonding interactions within the pocket as the closest residues are outside of van der Waals radius. However, the additional oxygen atom of the phosphite, to which it is equivalent, points towards the D206/R178 salt bridge to one side of the HtxB binding pocket. This phosphite-oxygen atom is 2.8 Å away from the oxygen atom of D206 and 3.2 Å away from the nitrogen atom of R178, suggesting that both residues interact with this phosphite-oxygen, albeit with a rather long hydrogen bond to the arginine (Fig. 2D). Although we could not directly observed the position of the hydrogen atoms in the structure, at pH 5 it is likely that the majority of the phosphite in solution is mono-anionic (p*K*_a_ 1.3, 6.7, Fig. 1), carrying a single proton on one of the three oxygen atoms. The 2.8 Å distance between the phosphite-oxygen and D206 implies, therefore, that the phosphite is protonated at this position and acts as a hydrogen bond donor to D206. These additional hydrogen bonds appear to provide enough stabilising energy to trap a fully closed complex in the crystal structure, which is virtually identical to the conformation observed in the complex with hypophosphite (Fig. 2C) (RMSD C*α*: 0.25 Å). Above pH 6.7, phosphite is predominantly found as the di-anionic form, lacking an available proton to hydrogen bond with the aspartate, which is consistent with the very weak binding (*K*_d_ > 1 × 10^4^ *μ*M) observed between phosphite and HtxB in our previous MST assays at pH 7.4^24^.

## Binding of Phosphite to HtxB in Solution Shows a Distinct pH Dependence

In order to characterize the pH dependence of the binding event between HtxB and phosphite, we used hydrogen and phosphorus NMR to directly observe the interaction in solution. The covalent bond between P and H atoms in phosphite gives rise to a ^1^J_PH_ coupling which appears as a doublet in the ^31^P NMR spectra. This ^1^J_PH_ coupling can be used in conjunction with chemical shifts to characterize the protonation state of the phosphite, (for general NMR method see^25,26^) and can be removed by application of ^1^H decoupling during acquisition of ^31^P spectra, resulting in a single peak. The linebroadening of these single peaks is dominated by transverse relaxation of the nuclei in question, therefore, binding of phosphite to HtxB would increase the linewidth of observable NMR resonances as a protein-phosphite complex will tumble slower than free phosphite in solution, increasing the transverse relaxation of the phosphorous nucleus and enabling us to detect ligand binding.

**Figure 3:**
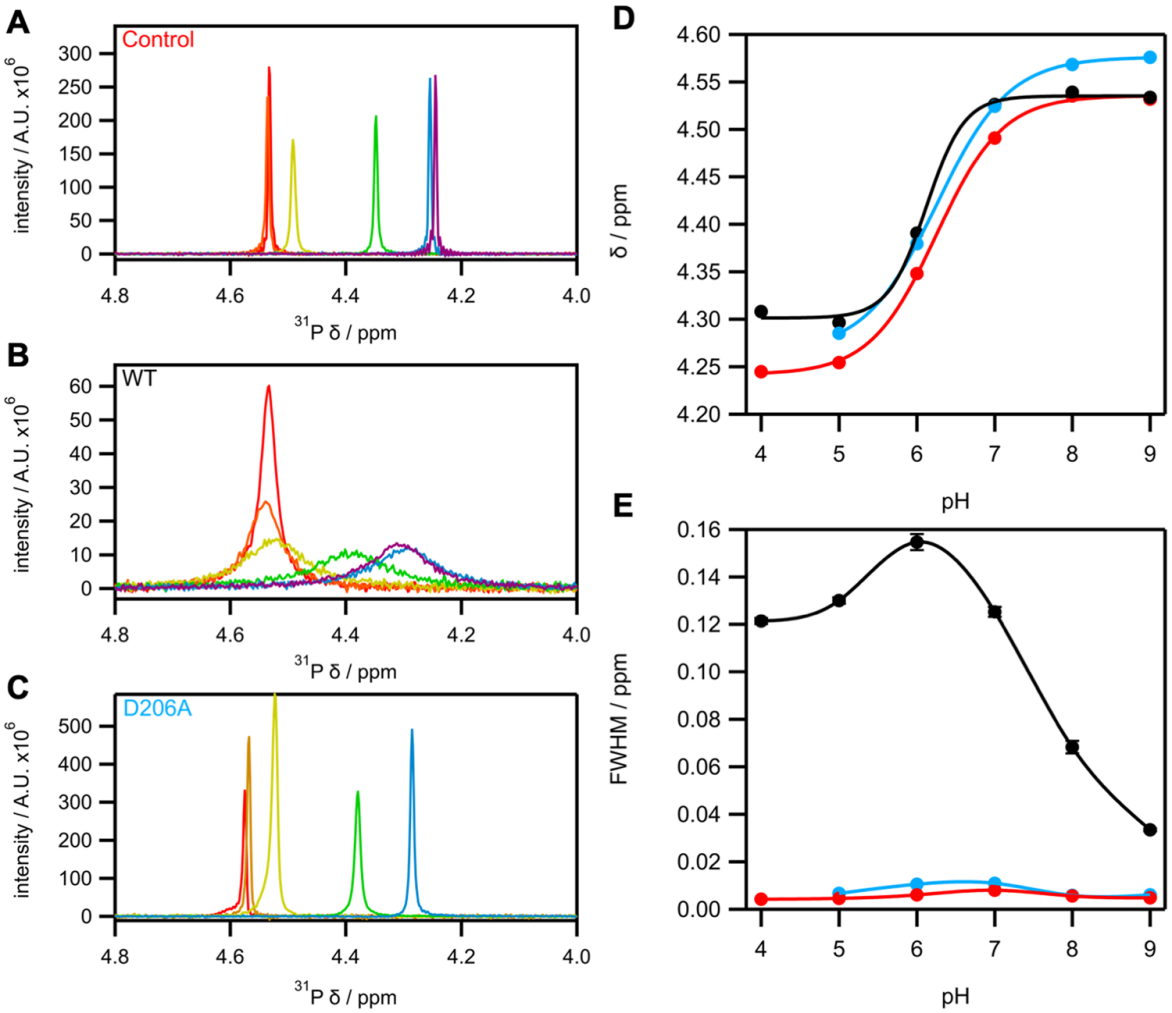
Expansion of ^31^P non-decoupled spectra of (**A**) 10 mM phosphite in the absence of protein, (**B**) 10 mM phosphite plus WT HtxB and (**C**) 10 mM phosphite plus D206A HtxB in 50 mM Tris-Acetate buffer, pH 9–4. Colors indicate pH of measurement: Red, 9; Orange, 8; Yellow, 7; Green, 6; Blue, 5; Violet, 4. (**D**) pH dependence of the ^31^P chemical shift of 10 mM phosphite in the absence of protein (red) and presence of WT HtxB (black) and D206A HtxB (blue). Chemical shifts were estimated by fitting peaks to a Lorentzian distribution (Eq. 3). The lines are theoretical and are fitted to a sigmoid relationship (Eq. 4), with calculated p*K*_a_ values reported in Table 2. (**E**) pH dependence of line broadening of 10 mM ^31^P in the absence of protein (red) and presence of WT HtxB (black) and D206A HtxB (blue). Full width at half-maximum (FWHM) estimated from fitting peaks to a Lorentzian (Eq. 3). Lines in (**E**) are theoretical cubic splines for guidance only.

In the absence of protein, we performed a pH titration of 10 mM sodium phosphite in 50 mM Tris-acetate buffer across the pH range 4–9 and followed it using both 1D ^31^P (Fig. 3) and 1D ^1^H NMR (Supplementary Fig. S1). We also followed ^31^P chemical shift changes and ^1^J_PH_ couplings across the pH titration, which resulted in a p*K*_a1_ value of 6.3 ± 0.1 (Fig. 3D,E and Table 2) compared to previously published values of p*K*_a1_ 6.2, 6.5 and 6.7^27–29^. Negligible ^31^P or ^1^H line-width changes were observed for the phosphite peaks across the pH titration (Fig. 3A and Supplementary Fig. S1). We repeated the pH titration in the same conditions but with the addition of 1 mM HtxB, recording both 1D ^1^H and ^31^P NMR spectra (Fig. 3B and Supplementary Fig. S1). The change in chemical shift of the non-decoupled ^31^P and ^1^H phosphite peaks indicated a p*K*_a_ of 6.2 ± 0.1, which closely matched the chemical shift change for free phosphite (Supplementary Fig. S1 and Table 2). An upfield shift of the decoupled ^31^P phosphite peak was observed as the pH of the buffer was decreased, which is in agreement with the control experiment in the absence of HtxB (Fig. 3B). Minimal chemical shift difference was observed for the phosphite peak on addition of HtxB, however significant linebroadening of the ^31^P peak was observed on reducing the pH from 9 to 4 (Fig. 3B). This observation indicates that phosphite is binding to HtxB in fast, approaching intermediate, exchange across this pH range, thus has weak binding affinity. The same linebroadening effect at lower pH values is a result of increased transverse relaxation rates that occur only when HtxB is present (Fig. 3E). Maximal line broadening is observed at pH 6, indicating the longest average residence time of phosphite in complex with HtxB is at a pH close to the p*K*_a_ of phosphite in solution (Fig. 3D, Table 2). Together, this shows that in solution, HtxB is capable of binding phosphite at pH values lower that 6, in agreement with the crystal structure, but that the binding affinity for phosphite is weak.

**Table 2:** p*K*_a1_ values of phosphite in the presence and absence HtxB determined via pH titration.

Experiment	Probe	p*K*_a1_
**10** **mM Phosphite control**		
*δ*	^31^P	6.26 ± 0.02
^1^J_PH_	^31^P	6.29 ± 0.02
^1^J_PH_	^1^H	6.30 ± 0.02
**1 mM HtxB + 10 mM Phosphite**		
*δ*	^31^P	6.18 ± 0.04
^1^J_PH_	^31^P	6.20 ± 0.03
^1^J_PH_	^1^H	6.22 ± 0.06
**450 ** ***μ*** **M HtxB + 10 mM Phosphite**		
*δ*	^31^P	6.26 ± 0.01
^1^J_PH_	^31^P	6.26 ± 0.01
^1^J_PH_	^1^H	6.26 ± 0.01

**Figure 4:**
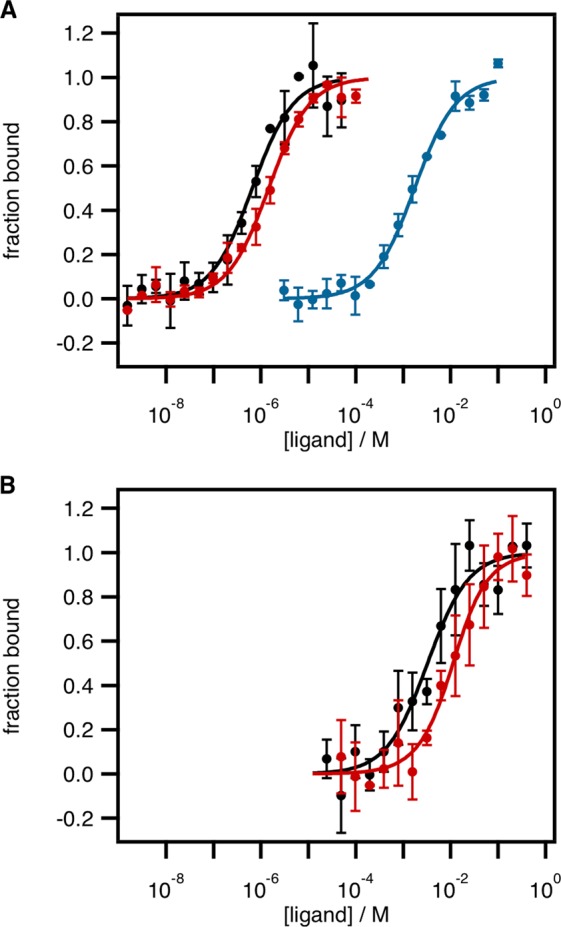
Thermophoresis determined binding affinities of WT HtxB and D206A HtxB for phosphite and hypophosphite. Binding curves for (**A**) HtxB WT and (**B**) HtxB D206A with hypophosphite (black, pH 7.4; red, pH 5) and phosphite (blue, pH 5). Proteins were labelled with NT-647-NHS dye (10 nM final concentration) and MST performed in 40 mM citrate-phosphate buffer, 250 mM NaCl, 0.05% Tween-20, pH 7.4 or pH 5, with proteins subject to 22 seconds of thermophoresis. As appropriate, labeled proteins were mixed with serial dilutions of hypophosphite or phosphite. Calculated dissociation constants, *K*_d_, are listed in Table 2. Error bars indicate the standard deviation from the mean of three independent titrations.

Having confirmed that HtxB is able to bind phosphite at pH values lower than 6 by NMR, we used Microscale Thermophoresis (MST) to determine a *K*_d_ for this interaction (Fig. 4 and Table 3). We labeled the protein with the amine reactive dye, NT-647-NHS, rather than the His-tag fluorescent label that we used in our previous study^24^, which cannot make a stable interaction with the protein at pH 5. A 1:1 ratio of protein molecule to dye was achieved via attachment to a surface exposed lysine group. We determined the *K*_d_ for HtxB binding to hypophosphite as 650 ± 130 nM (in 40 mM citrate-phosphate buffer, pH 7.4), compared to 560 ± 110 nM in 50 mM pH 7.4 HEPES buffer using the His-tag label (recorded previously^24^). Under the same citrate buffer conditions, no binding of phosphite to HtxB was detected up to 100 mM phosphite, but on adjusting the pH of the buffer to 5, HtxB binds phosphite weakly, with a *K*_d_ of 1.74 mM. The reduction of pH also results in an approximately two-fold reduction in binding affinity for hypophosphite, with a recorded *K*_d_ value of 1.46 *μ*M. Thus, even though HtxB can be crystallised in a closed conformation with a large excess of phosphite at pH 5, the MST results corroborate the NMR experiments, demonstrating that the binding affinity for the ligand in solution is weak.

**Table 3:** Calculated *K*_d_ values for ligands binding to WT HtxB and D206N/D206A HtxB mutants at pH 7.4 or pH 5 (NBD = no binding detected up to 100 mM ligand).

Protein	Hypophosphite (*μ*M)		Phosphite (*μ*M)	
	pH 7.4	pH 5	pH 7.4	pH 5
WT	0.65 ± 0.13	1.46 ± 0.16	NBD	1.74 × 10^3^ ± 0.221 × 10^3^
D206A	3.24 × 10^3^ ± 0.93 × 10^3^	7.76 × 10^3^ ± 5.07 × 10^3^	NBD	NBD
D206N	NBD	NBD	NBD	NBD

## The effect of D206A and D206N Mutations on Ligand Binding in HtxB

To probe the role of the D206 in ligand binding we generated D206A and D206N mutants of HtxB. Both proteins were produced and purified using the same methods as for WT HtxB. The mutations produced no gross changes in the secondary structure content of the protein, as observed by CD spectroscopy (Supplementary Fig. S2). MST assays show that in the presence of 20 mM acetate the D206A mutant binds hypophosphite at both pH 7.4 and pH 5, but with a *ca*. 5000 × reduction in binding affinity compared to the WT protein in the same conditions (Fig. 4 and Table 3). When we assayed D206A HtxB in acetate-free conditions at pH 7.4, no binding of hypophosphite could be detected up to 400 mM of ligand (data not shown). No evidence of D206A HtxB binding to phosphite at either pH 7.4 or 5 could be detected and ^31^P NMR spectra of D206A HtxB in the presence of 10 mM phosphite did not show significant linebroadening of the phosphite peak across the pH range 4–9 (Fig. 3C). The D206N mutation renders D206N HtxB incapable of binding phosphite or hypophosphite at pH 5 or 7.4, as determined by MST with up to 100 mM of ligand (Table 3).

**Figure 5:**
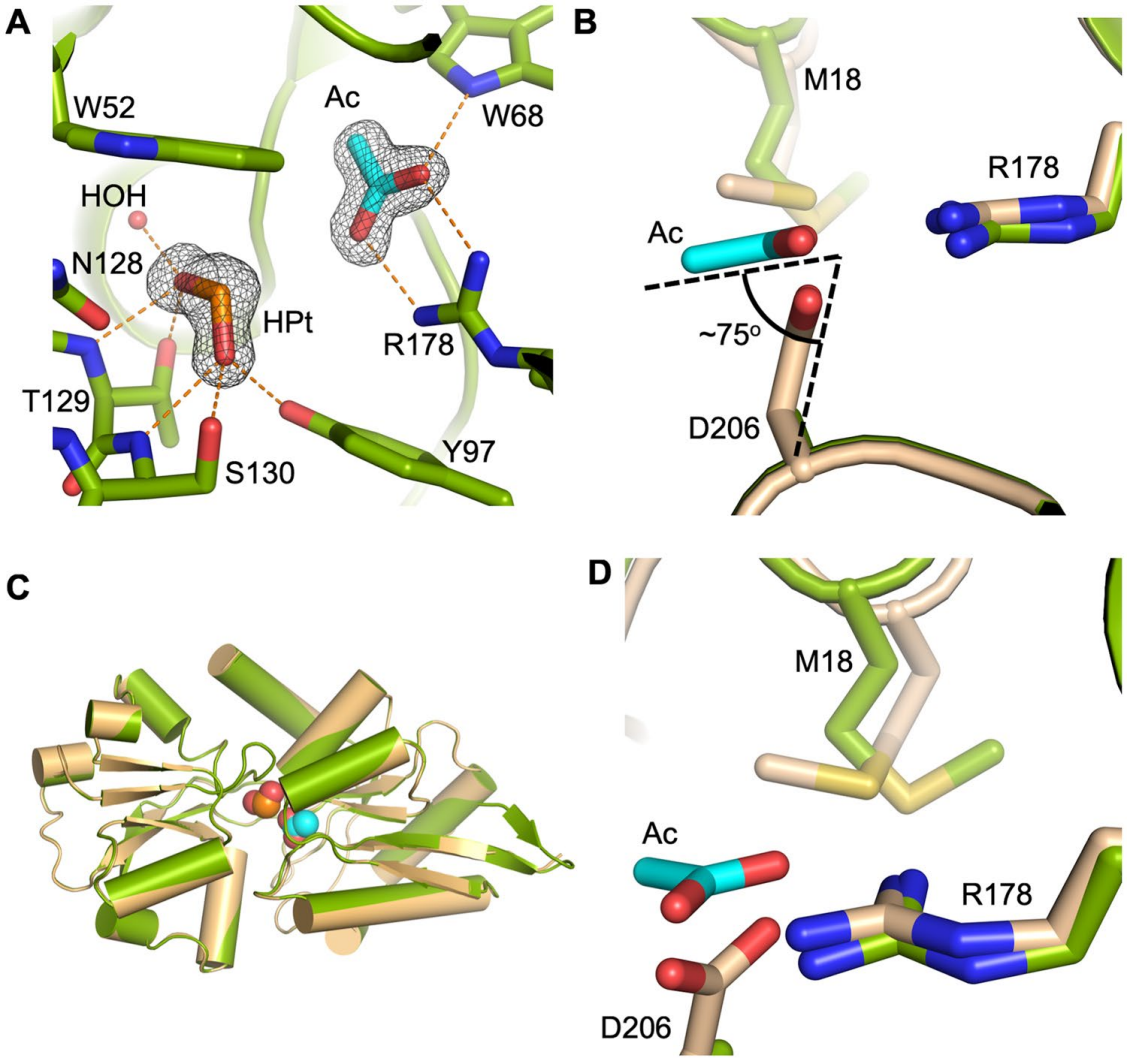
An acetate molecule fulfills the role of D206 in the structure of D206A HtxB in complex with hypophosphite. (**A**) Acetate (cyan) forms a salt bridge with R178 and hydrogen bonds to W68 in the D206A complex with hypophosphite (green). Omit maps (black mesh, contoured at 1.2 *σ*) are shown around the hypophosphite and the acetate molecules. Hydrogen bonds are shown as orange dashes and a single water molecule (HOH) is shown as a red sphere. A superposition of D206A HtxB (green) and WT HtxB (orange), both in complex with hypophosphite, shows that the fold and the degree of domain closure is identical. (**B**) The acetate (cyan) sits in the plane of the guanidinium group of R178, unlike D206 from the WT structure (beige), which lies 1 Å below the plane at and angle of 75° (shown as black dashed lines). (**C**) A superposition of WT HtxB (beige) and D206A HtxB (green) in complex with hypophosphite with the ligands shown as spheres. (**D**) The change in rotamer conformation of M18 between the WT and D206A structures.

D206A HtxB was crystallised in complex with hypophosphite (PDB:6GHT) in a closed conformation that was equivalent to the WT complex (see Table 1 for data collection and refinement statistics). In the structure, the extra space generated by the alanine mutation allows for a single molecule of acetate to fulfill the role of D206 in the WT protein, forming two hydrogen bonds to R178, and a further hydrogen bond to the indole ring-nitrogen of W68 (Fig. 5A). We cannot account for the origin of the acetate, but this explains why we only detect binding of hypophosphite to D206A HtxB via MST in acetate containing buffers. All of the atoms of the acetate molecule lie in plane of the guanidinium group of the arginine, forming a classic end-on salt bridge interaction. This differs from the direction of approach of the aspartate to the arginine in the WT complex, where the two oxygen atoms of the aspartate lie 1 Å below the plane of the guanidinium group, with the C*α*-C*β* bond of D206 offset by approximately 75° from the same plane (Fig. 5B). It is not possible to say from the electron density map or interactions with the surrounding residues if the acetate is protonated at the oxygen that points towards the hypophosphite, but the crystallisation pH is less than the *pK*_a_ of acetate (4.76)^30^. The mode of binding of the hypophosphite and the degree of domain closure is the same as the WT protein (RMSD C*α*: 0.19 Å) (Fig. 5C), but the binding of the acetate slightly disrupts the local packing around the binding pocket, causing a change in rotamer conformation of M18 (Fig. 5D). Clearly, the high concentration of hypophosphite in the crystallisation experiment along with the presence of acetate favours the formation of a closed, ligand-bound complex of D206A HtxB, that would not readily form in solution, given the weak binding affinity for ligand. However, the observation of acetate binding to facilitate the formation of the buried salt bridge in the closed complex of D206A HtxB, suggests that this intramolecular interaction is a prerequisite for capture of the ligand.

**Figure 6:**
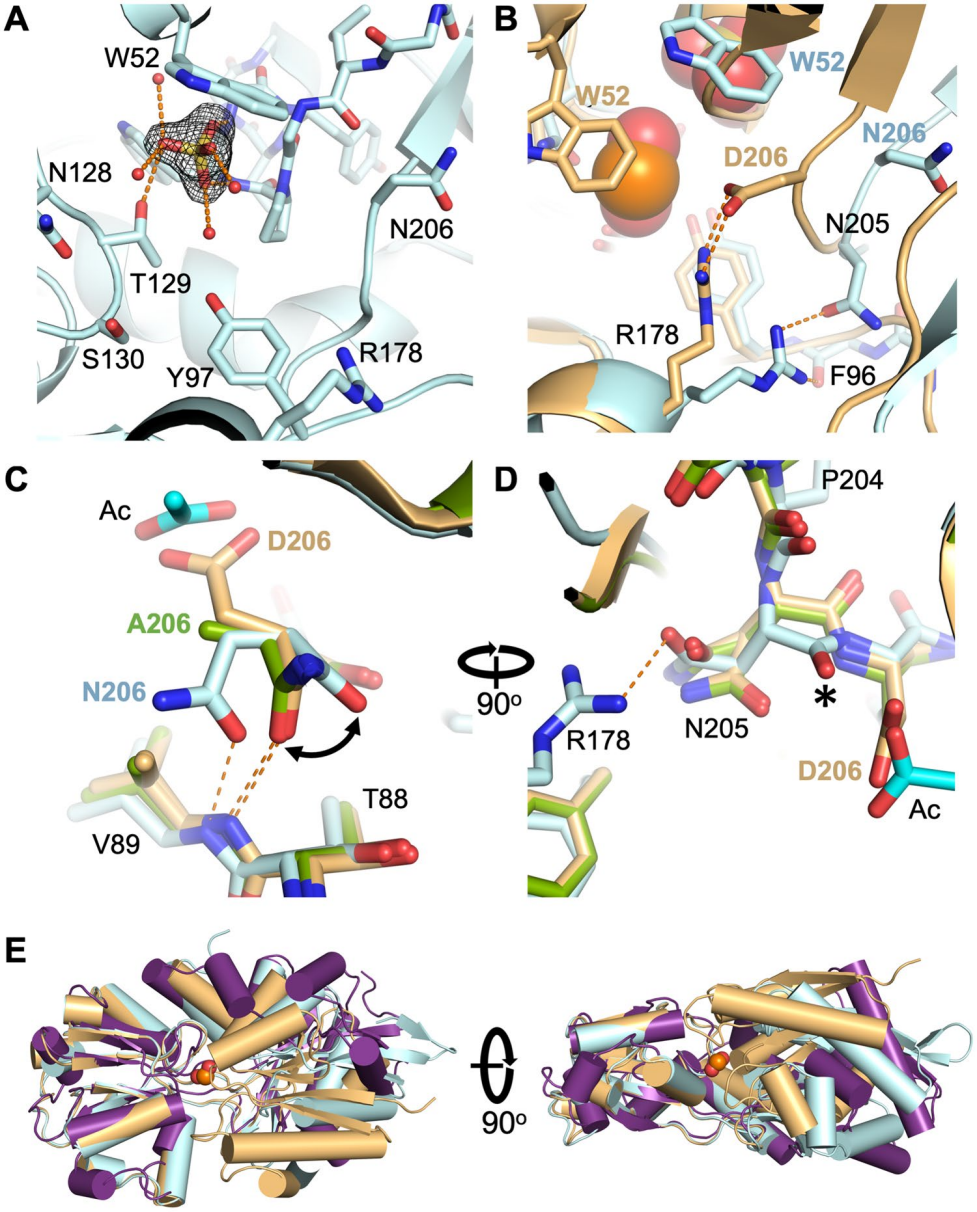
D206N HtxB crystallises in a partially open complex with sulphate. (**A**) The central cleft of D206N HtxB (blue), showing the location of a sulphate molecule, which binds at the N-terminal end of helix 3, forming hydrogen bonds with the mainchain amides of P71, W72 and G73, the sidechain of T129 and four water molecules. An omit map surrounding the sulphate moiety (black mesh, contoured at 1.5 *σ*) is also shown. (**B**) A comparison with WT HtxB in complex with hypophosphite (beige) shows the spatial relationship of the two ligand binding sites (ligands shows as spheres) and the difference in the position of the binding pocket residues in the mutant versus the WT protein. Hydrogen bonds are represented as orange dashed lines. (**C**,**D**) A comparison between the D206N HtxB (blue), D206A HtxB (green) and WT HtxB structures (beige) (superimposed on the large domain: N-F96 and P204-C) shows how the D206N mutation changes the hydrogen bonding across the beta-sheet of this domain. In the WT and D206A HtxB structures the carbonyl of residue 206 hydrogen bonds with the mainchain amide of V89 (orange dashes, 3.0 Å). In the D206N HtxB structure, the sidechain oxygen of N206 sits within hydrogen bonding distance (2.9 Å) of the V89 amide and the N206 carbonyl is rotated by 50° (black arrow). The N205 carbonyl flips 180° (black asterisk), altering the rotamer of N205 in the D206N HtxB structure and resulting in a new hydrogen bond being formed with R178. The acetate molecule from the D206A HtxB structure (cyan) is shown for reference. (**E**) Two orthogonal views of superimposed HtxB-like proteins in closed (HtxB WT with hypophosphite; beige), partially-open (HtxB D206N mutant with sulphate; blue) and fully open (apo-PtxB from *Pseudomonas stutzeri*; purple) conformations. The superposition is carried out on the smaller of the two domains (residues 99–200 in HtxB), containing the important loop that recognises the oxygen atoms of the cargo (HtxB; N128-S130, PtxB; S124-S126).

D206N HtxB crystallised in a partially open conformation (PDB:6GHQ, crystallised in the presence of hypophosphite) (see Table 1 for data collection and refinement statistics) in complex with a sulphate molecule that binds at the N-terminal end of helix 3, presumably being partially stabilised by the helix dipole at this location (Fig. 6A). Both sulphate and phosphate could be refined into the electron density map at this position, but the crystallisation conditions contained 0.2 M sulphate and we determined that phosphate binding to D206N HtxB was incredibly weak, therefore it was unlikely to co-purify (Supplementary Fig. S3). The sulphate binding site is approximately 6 Å (S-P distance) away from the native hypophosphite binding site in the WT HtxB structure (Fig. 6B), forming hydrogen bonds with a number of surrounding water molecules (Fig. 6A), including the mainchain amides of P71, W72, G73, and the sidechain of T129, which is one of the conserved binding pocket residues required for hypophosphite binding. A comparison of the D206N HtxB structure with the closed structure of WT HtxB (PDB:5ME4) and the open structure of apo *P. stuzeri* PtxB (PDB:5O2K)^24^, using DynDom^31^, shows that one domain of D206N HtxB is rotated by 35° relative to the other around an axis that lies between the two domains, which is approximately half of the rotation observed between the closed and open complexes (60°) of PtxB (Fig. 6E). This shows that D206N HtxB crystallises in a partially open conformation. Considering the inability of D206N HtxB to bind phosphorus ligands in solution, along with the observation of this partially open confirmation, this suggests that the D206N mutant is unable to achieve a closed, ligand-bound state.

Superposition of the ligand binding domain (residues 95–204) from D206N HtxB with WT HtxB (Fig. 6B) shows that N206 points away from the ligand binding site in the mutant protein, rotated *ca*. 180° away from its position in the closed WT complex, and therefore, not in a position where it can interact with R178. We note that residue 206 sits on one of the two hinge regions of the protein (F96-K98 and P204-N206), suggesting it may have some flexibility. Closer comparisons of the D206N, D206A and WT HtxB structures (Fig. 6C,D), focusing on the conformation of the larger domain (superposition of the N-terminus to F96 and P204 to the C-terminus) shows key differences in the interactions that residue 206 and its neighbours make with the surrounding polypeptide. In the WT and D206A HtxB structures the carbonyl of residue 206 hydrogen bonds with the mainchain amide of V89 (3.0 Å), as part of the hydrogen bonding network across the anti-parallel beta-sheet in this domain. In the D206N HtxB structure, the sidechain oxygen of N206 sits within hydrogen bonding distance of the V89 amide (2.9 Å), altering the mainchain bond angles and rotating the N206 carbonyl by 50° away from its position in the WT/D206A HtxB structures. This change in mainchain conformation results in a 180° flip in the N205 carbonyl, requiring N205 to adopt a different rotamer in the D206N structure and leading to a new hydrogen bond formed with R178. R178 itself, also forms a hydrogen bond with the mainchain carbonyl of F96 (2.9 Å), which also sits in the hinge region. It seems likely that this rearrangement of the mainchain very close to the hinge region, together with the concomitant change in the hydrogen bond network across the domain interface might result in the D206N HtxB mutant being unable to form a closed conformation in complex with phosphorus ligands, explaining why no ligand binding could be detected for this mutant in our solution studies.

## Discussion

PBPs exist in a state of dynamic equilibrium between open and closed conformations, facilitated by a highly flexible hinge region^32,33^. When a specific ligand is bound to the protein, a closed state is favoured and stabilised to allow for cargo to be shuttled to the transporter in the inner membrane. We have shown that HtxB is capable of binding phosphite at pH 5 due to a change in the protonation state of the ligand that facilitates the formation of extra hydrogen bonds within the binding pocket of the protein, enabling the formation of a closed complex. Although this complex can be captured by crystallisation with a large excess of phosphite (5 mM), the *K*_d_ of HtxB for phosphite at pH 5 is only 1.74 mM, which is *ca*. 1000 × less than hypophosphite at the same pH. At neutral pH we could detect no binding of phosphite to HtxB, suggesting that HtxB is not a good phosphite transporter in conditions equivalent to those found in nature. In our structure at pH 5, the protonated phosphite forms a hydrogen bond with both D206 and R178. In complex with hypophosphite, neither of these residues form a direct interaction with the ligand, but instead form an intramolecular salt bridge across the domain interface. This generates one wall of the ligand binding pocket, delimiting a space adjacent to the ligand. Based on the much weaker binding of phosphite to HtxB at neutral pH, we therefore predict that binding of di-anionic phosphite would generate a sub-van der Waals distance between the third oxygen position and the D206/R178 salt bridge, producing a steric clash that would prevent closure of the domains around the ligand and lead to subsequent ligand release. It is only by prototonating the oxygen at this position by reducing the pH to below 6 that it can act as a hydrogen bond donor, forming a stabilising interaction with the residues of the salt bridge. However, given the weak binding, it is likely that the off-rate of the ligand is very rapid. Perturbation of this salt bridge by introducing a D206A or D206N mutations completely disrupts hypophosphite or phosphite binding to HtxB at pH 7.4 or 5. It is only by the addition of acetate that partial binding of hypophosphite can be restored in the D206A mutant, but with a 5000 × reduction in binding affinity compared to WT. This further suggests that the formation of the intramolecular salt bridge between D206 and R178 is critical for HtxB to bind ligands. Comparisons of the D206N structure, which crystallised in a partially open conformation, with open and closed complexes from closely related proteins suggests that D206 and R178 are positioned on flexible regions of the protein that are brought into close proximity during domain closure. This suggests that the residues surrounding the binding pocket are arranged in such a way that binding of the correct ligand is a requirement for domain closure; this is in addition to the selection of a P-H bond by the P-H…*π* interaction with the capping aromatic residue.

**Figure 7:**
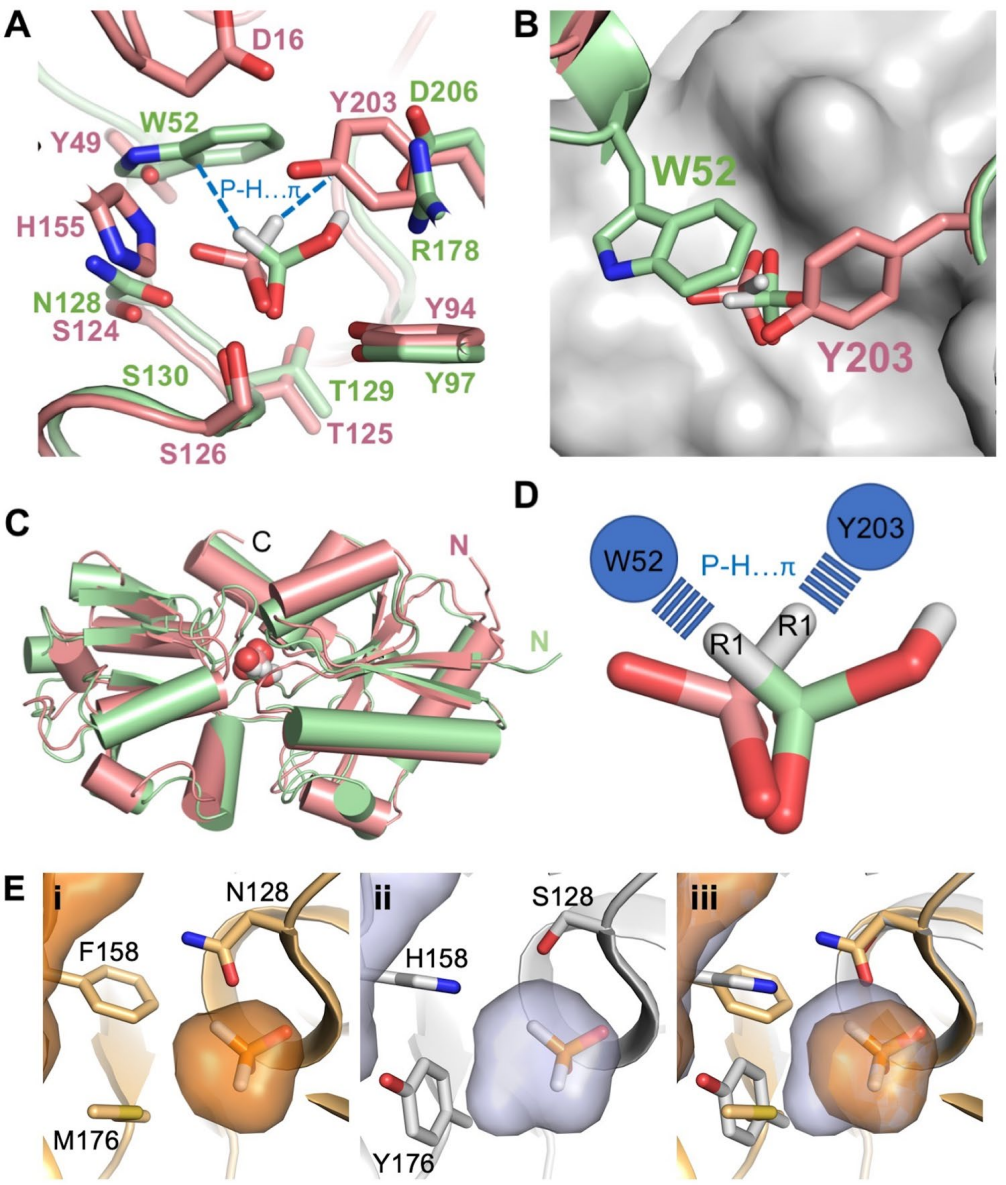
Sequence differences in the binding pocket of related phosphite and hypophosphite PBPs change the shape and orientation of the ligand binding site. (**A**) Sequence conservation in the binding pocket and mode of binding of phosphite in HtxB (green) and PtxB (pink). The protein is drawn as a cartoon, with the ligands and side chains drawn as sticks. The P-H…*π* interaction is drawn as a blue dashed line. (**B**) A view of the relative orientation of the *π* system that provides ligand specificity. The view is rotated 90° relative to (**A**), with the positions of W52 and Y203 shown as sticks above the phosphite. The molecular surface of the binding pocket is shown in grey. (**C**) The fold and conformation of the two proteins is largely conserved. The protein backbones are represented as a cartoon and the ligands as spheres. (**D**) The mode of binding of phosphite in HtxB and PtxB are mirror-images of each other. The phosphite molecules are shown as sticks and the relative position and interactions with W52 and Y203 are indicated schematically with circles and dashed lines, respectively. (**E**) The volume of the binding pocket (shown as an enclosed surface) in (i) HtxB (orange), (ii) modelled HtxB-hybrid (grey) and (iii) an overlay of the two volumes. Sequence differences around the binding pocket generate more space in the hybrid. The position of hypophosphite in the HtxB structure is shown for reference.

PBPs that have evolved to bind phosphite, hypophosphite and other reduced phosphorus compounds are structurally and sequentially closely related. The mode of binding of phosphite in complex with HtxB can therefore be directly compared with that of phosphite in the phosphite-binding PBP, *P. stutzeri* PtxB (PDB: 5O2J). This shows how an alternative positioning of the *π* system, which confers ligand specificity in both proteins (W52 in HtxB compared to Y203 in PtxB), to opposite sides of the binding pocket dictates that the R1 hydrogen of the phosphite must point in different directions (Fig. 7A,B). Thus, two of the phosphite-oxygen atoms have a conserved position in each protein, with the phosphite rotated *ca*. 180° around an axis that bisects the phosphorus center and lies equidistant between the two common oxygen atoms. The effect of this is that the ligands are bound as mirror-images of each other (Fig. 7D) and is an example of how subtle changes of sequence on a similar molecular scaffold can influence specificity dependent on the relative geometric positions of interacting atoms in the ligand.

Binding affinities for phosphite and hypophosphite in the nanomolar range are required in this family of transporter because ligand availability in natural environments is very low. We could not detect binding of phosphite to HtxB at neutral pH, which suggests that phosphite uptake is unlikely to be sustained solely by the HtxBCDE transporter in typical aqueous conditions. However, recent work by Motomura *et al*.^15^, building on previous work from the same group in *E. coli*^13^, shows how the HtxBCDE transporter is capable of sustaining growth in supplemented phosphite conditions of a strain of the cyanobacteria, *Synechococcus elongatus*, that had been genetically modified to lack any alternative phosphorous import systems. The modified strain could not survive in simulated pond or fresh water media containing no phosphite, but could out-compete the WT strain in conditions containing 0.2 mM phosphite, whilst in the presence of 0.2 mM phosphate, the WT strain dominated the cultures and the modified strain perished. This conferred phosphite-dependency has therefore been proposed as a potential mechanism for biological containment of genetically engineered organisms. Based on the binding data and structures reported here, we suspect that the observed phosphite uptake by HtxB could be due to a slight acidification of the growth media under the experimental conditions used, enabling uptake of mono-anionic phosphite at concentrations consistent with the *K*_d_ of 1.74 mM that we recorded. We propose that uptake of phosphite is likely to be better at a pH less than 6, which might allow growth on lower phosphite concentrations.

More broadly, we have shown that subtle changes to the binding site of HtxB (the difference of one or two hydrogen bonds) decrease binding affinity of ligands by 2–3 orders of magnitude, thus it is likely that specific point mutations around the third oxygen site could, with very subtle structural changes, increase the binding affinity of HtxB for phosphite. In an attempt to explore this further we have modelled the binding pocket from a sub-family of “HtxB-like” proteins from the soil-dwelling bacteria *Bradyrhizobium sp. BTAi1*, *Methylopila sp. 73B* and *Lutibaculum baratangense AMV1* (*ca*. 50% sequence identity to HtxB), which also share some sequence similarities with PtxB around the ligand binding pocket. We have identified three positions around the ligand binding site that vary between HtxB and the HtxB-like proteins, which are N128 to serine (also serine in PtxB), F158 to histidine (also histidine in PtxB) and M176 to tyrosine or phenylalnine (serine in PtxB) (Fig. 7E). Each of these residue differences generate extra space in the binding pocket of the HtxB-like protein model, resulting in an extra 20 Å^3^ of volume, compared to HtxB (model, 88 Å^3^; HtxB, 68 Å^3^, calculated with CASTp^34^), plus, the extra histidine moiety provides a hydrogen bond donor/acceptor within proximity to the ligand, suggesting that in the HtxB-like proteins, this histidine is likely to carry out a similar role as in PtxB, forming an interaction with bound ligand. As the pocket is both bigger, with extra capacity for hydrogen bonding, and still possesses the tryptophan-mediated P-H…*π* cap, it is likely, based in the understanding gained from this study, that it can bind phosphite. With further investigation, there could be scope to optimise binding of phosphite, while maintaining the exclusion of phosphate, to HtxB or this family of HtxB-like proteins, to optimise these transporters for bioengineering and biocontainment strategies using phosphite.

## Methods

### Protein preparation

The QuickChange II Site-Directed Mutagenesis Kit (Agilent Technologies, USA) and primer pairs D206N forward (5′-gttcgaaacttccgaacaacgccattagtgtacca-3′) and D206N reverse (5′-tggtacactaatggcgttgttcggaagtttcgaac-3′) or D206A forward (5′-ttcgaaacttccgaacgccgccattagtgtaccaa-3′) and D206A reverse (5′-ttggtacactaatggcggcgttcggaagtttcgaa-3′) were used to generate plasmids encoding D206N and D206A variants of HtxB. The mutated genes were verified by automated DNA sequencing (GATC Bioech). WT, D206N and D206A HtxB proteins were produced and purified as described previously^24^.

### X-ray crystallography

5 mM sodium phosphite or sodium hypophosphite was added to HtxB or each of the HtxB D206N/A mutants (10 mg/ml) and crystallised by sitting-drop vapor diffusion at 290 K (50 *μ*l drop and 50 *μ*l reservoir) using a Mosquito LCP crystallisation robot equipped with a humidity controlled stage (TTP Labtech) and commercial screens (Qiagen Nextal PACT and JCSG+). WT HtxB with phosphite crystallised in conditions containing 0.2 M magnesium chloride, 0.1 M Na acetate pH 5 and 20% (w/v) PEG 6000. D206N HtxB was crystallised in 0.2 M ammonium sulphate, 0.1 M Bis-Tris pH 5.5 and 25% (w/v) PEG 3350. D206A HtxB was crystallised in complex with hypophosphite in 0.1 M Bis-Tris pH 5.5 and 25% (w/v) PEG 3350. A single crystal of each was harvested and cryoprotected in its mother-liquor with an additonal 25% ethylene glycol prior to plunge-cooling with liquid nitrogen. Crystals were mounted (100 K) on beamlines i04-1, i04 and i03, at the Diamond Light Source, respectively, and data were collected at wavelengths of 0.92819 Å, 0.97951 Å or 0.9718 Å, respectively. Data were processed using the Xia2 pipeline^35^, which showed that the crystals belonged to either the spacegroup P212121 (WT and D206A HtxB) or P21212 (D206N HtxB) (See Table 1 for data collection statistics and cell dimensions). The structures were determined by molecular replacement with Phaser^36^ using a monomer of HtxB (PDB:5ME4) as a search model. Model building was carried out in Coot^37^, refinement in Refmac5 (ccp4i)^38,39^ and superposition with Superpose^40^ (See Table 1 for refinement and validation statistics). Structural validation was carried out with Molprobity^41^. Coordinates and structure factors are deposited in the Protein Data Bank under accession codes 6EMN, 6GHT and 6GHQ.

### NMR

One dimensional ^1^H and ^31^P spectra to characterize the pH dependent binding between HtxB and phosphite were acquired using a Bruker 500 MHz Avance spectrometer equipped with a 5 mm broadband probe tuned to 202.45631 MHz for phosphorous, and 500.130 MHz for proton. A spectral width of 50 ppm centered at 0 ppm enabled the observation of the relevant phosphorous signals, while a spectral width of 25 ppm centered at 4.7 ppm for ^1^H enabled the observation of all ^1^H resonances.

^1^H spectra were acquired using ^1^H presaturation and a Hahn-Echo refocusing pulse prior to acquisition. Spectra were typically accumulations of 256 transients with an acquisition time of 0.65 s. Both decoupled and un-decoupled ^31^P spectra were acquired using *zgig* and *zg* pulse programs respectively from the Bruker library. Spectra were typically accumulations of 512-1024 transients with a 10 s inter-scan delay and a 0.81 s acquisition time. Both ^1^H and ^31^P NMR spectra were processed using an exponential window function and 5 Hz linebroadening. A 100% D_2_O capillary with 200 mM phosphate in HEPES(K^+^) buffer at pH 7.2 was included in the 5 mm NMR tube for the ^31^P experiments recorded for HtxB, to act both as a reference, and as a quality control measure for the shimming of the probe.

### Microscale Thermophoresis

20 *μ*M protein was labeled with a two-fold excess of NT-647-NHS dye (NanoTemper Technologies, Munich Germany), following the manufacturer’s instructions. Protein had a degree of labeling of 1 dye molecule per protein molecule. Binding affinity of proteins for ligands was determined using Microscale Thermophoresis (MST) with a Monolith NT.115 instrument (NanoTemper Technologies, Germany). 10 *μ*L of 20 nM labelled protein was mixed with 10 *μ*L of ligand in 40 mM citrate-phosphate, 250 mM NaCl, 0.05% Tween-20 pH 7.4 or 5.4 *μ*L of protein-ligand mixture was loaded into “Premium Grade Capillaries” (NanoTemper Technologies) and thermophoresis was measured at 22 °C for 22 s with 40% LED power and medium thermophoresis power. Data from three independent measurements were combined and analysed using the MO.Affinity Analysis software version 2.1 (NanoTemper Technologies), fitted to a single binding site model (Eq. 1) where U is the unbound normalised fluorescence, B is the fully bound fluorescence, [l] is concentration of ligand. Data were plotted using Igor Pro version 7.05 (Wavemetrics Inc., USA).

1 $$f(l)={\rm{U}}+\frac{({\rm{B}}-{\rm{U}})\times [l]+[{\rm{HtxB}}]+{K}_{{\rm{d}}}-\sqrt{{([l]+[{\rm{HtxB}}]+{K}_{{\rm{d}}})}^{2}-4\times [l]\times [{\rm{HtxB}}]}}{2\times [{\rm{HtxB}}]}$$

### CD spectroscopy

Protein (0.1 mg ml^−1^) was buffer exchanged into 5 mM sodium phosphate buffer, pH 7.4 and spectra were recorded in a cuvette with a 0.1 cm path length at 25 °C using a JASCO-810 spectrometer (JASCO, UK). Spectra were recorded continuously from 250 to 190 nm (50 nm s^−1^, 1 nm increments, 4 s response, 6 accumulations) and background subtracted before calculation of mean residue elipticity (MRW) using Eq. 2.

2 $${[\theta ]}_{{\rm{MRW}}}=\frac{\theta \times 100}{l\times N\times c}$$

### NMR figures

NMR spectra were output as ASCII files and represented in Igor. Chemical shifts and line broadening was estimated by fitting peaks to a Lorentizian function (Eq. 3).

3 $$f(x)={y}_{0}+\frac{A}{{(x-{x}_{0})}^{2}+B}$$

where *y*_0_ is the baseline, *A* is the peak amplitude, *x*_0_ is the center of the peak and *B* is the Full Width Half Maximum.

p*K*_a_ values were calculated by fitting Eq. 4 to either chemical shift data from ^31^P decoupled data, or coupling constants from ^1^H and ^31^P 1D spectra of phosphite peaks.

4 $$f(x)={\rm{base}}+(\frac{{\rm{\max }}}{(1+{10}^{(\frac{{{\rm{pK}}}_{a}-x}{{\rm{rate}}})})})$$

## Supplementary information

Supplementary Information

## Data Availability

The atomic coordinates and structure factors are available via the PDB under accession codes PDB: 6EMN, 6GHT and 6GHQ.
