# Supplementary material for: Phosphite binding by the HtxB periplasmic binding protein depends on the protonation state of the ligand
Source: Sci Rep. 2019 Jul 15;9:10231. doi: 10.1038/s41598-019-46557-2 (PMC6629693; doi:10.1038/s41598-019-46557-2)
Supplement: Supplementary file 1 — Supplementary Information [file 41598_2019_46557_MOESM1_ESM.pdf]

Supplementary Information

Phosphite binding by the HtxB periplasmic binding protein depends on the protonation state of the ligand

Nathan B.P. Adams, Angus J. Robertson, C. Neil Hunter, Andrew Hitchcock and Claudine Bisson

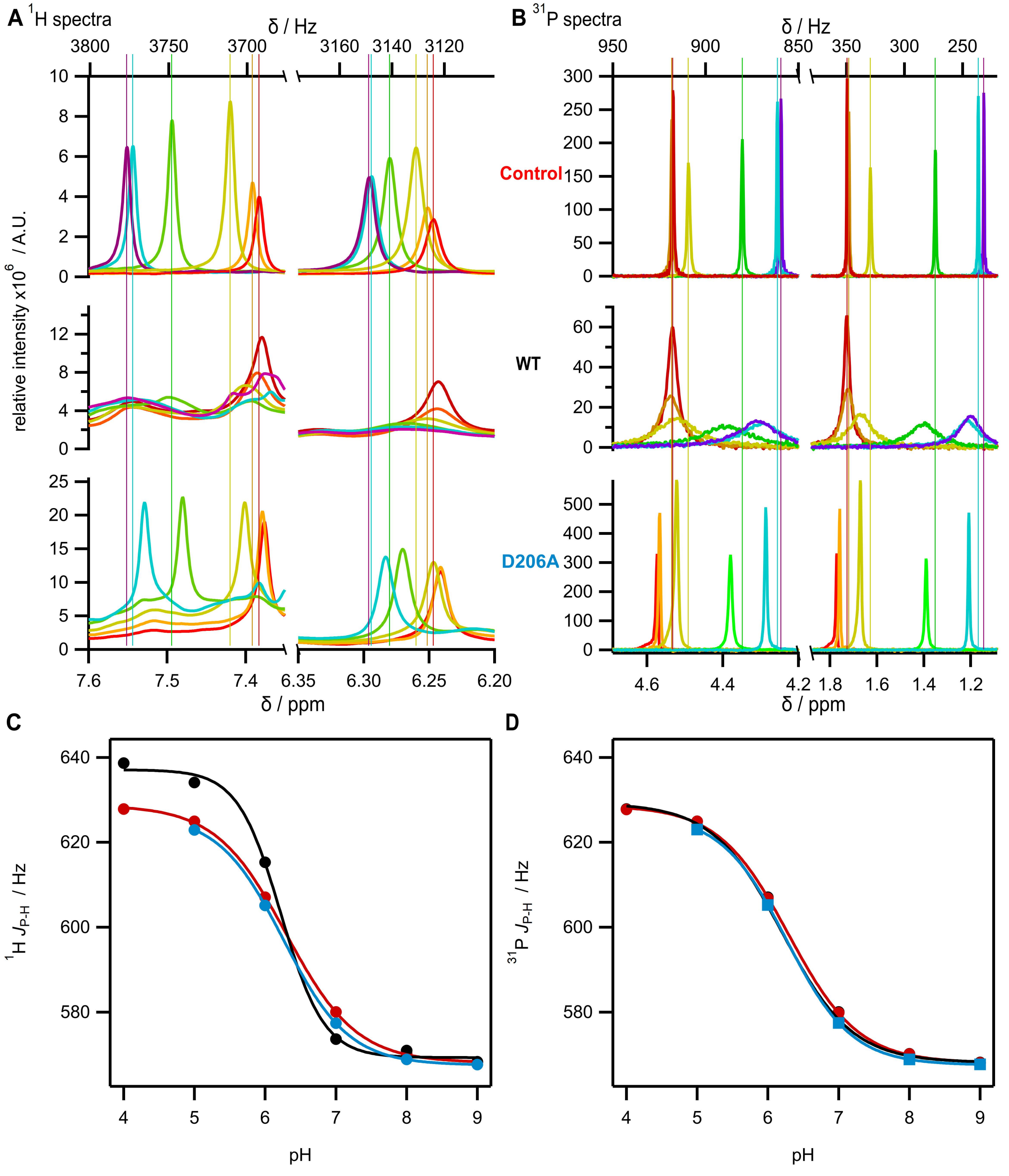

Figure S1. ^{1}H and ^{31}P spectra and coupling constant data from NMR pH titrations. (A) ^{1}H and (B) ^{31}P spectra of phosphite control, HtxB WT with 10 mM phosphite, and HtxB D206A, showing regions measured for coupling constants. Colors indicate pH of measurement: Red, 9; Orange, 8; Yellow, 7; Green, 6; Blue, 5; Violet, 4. pH dependence of $^1J_{HP}$ coupling constants; (C)^{1}H reporting and (D) ^{31}P reporting. Red, no protein; black, HtxB WT; blue, HtxB D206A. The lines are theoretical and are fitted to a sigmoid relationship (equation 4), with calculated pK_a values reported in table 2.

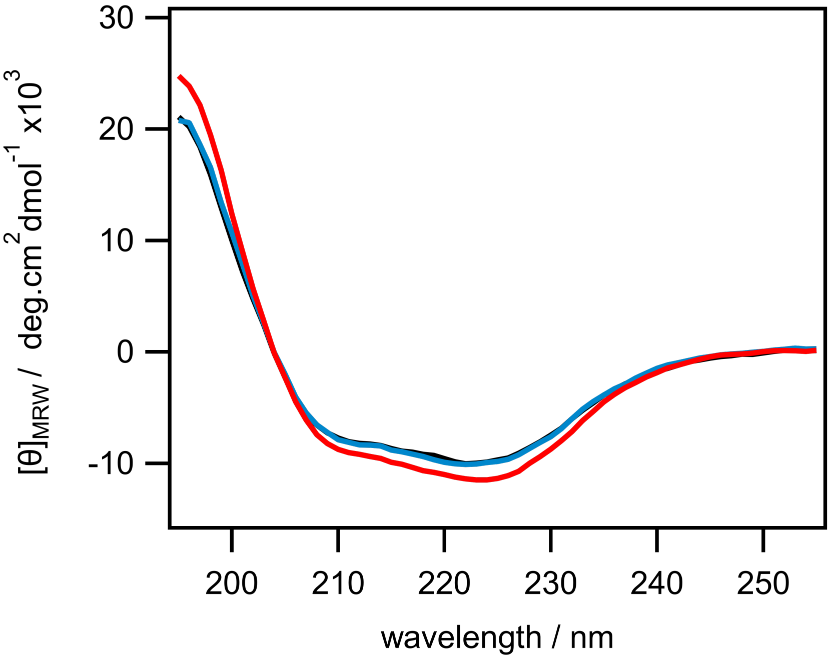

Figure S2. D206A and D206N mutations cause no gross changes in the secondary structure of HtxB. CD Spectra (mean residue elipticity) measured at 25 °C in 5 mM sodium phosphate, pH 7.4. Black, WT; blue, D206A; red, D206N.

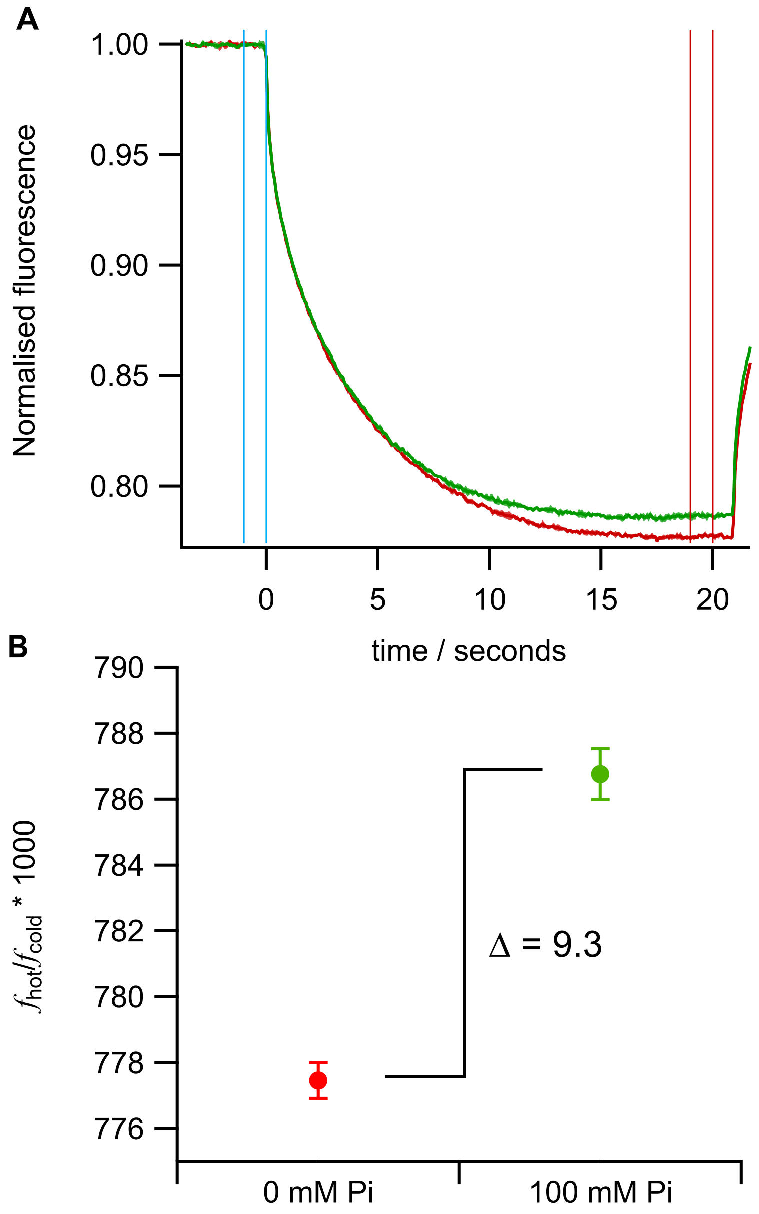

Figure S3. HtxB D206N binds phosphate weakly. (A) Normalized thermophoresis traces of HtxB D206N subjected to 22 seconds of thermophoresis in 50 mM HEPES pH 7.4, 250 mM NaCl, 0.05 % Tween-20 (red), and mixed with either 100 mM phosphate (green). Lines are mean fluorescence of 4 independent measurements (standard deviation shown by shading). Blue and red vertical lines indicate the time periods where the average of f_{hot}/f_{cold} ratio is calculated. (B) Category plot highlighting the difference in thermophoresis between apo-HtxB and HtxB mixed with 100 mM phosphate. The difference between plus and minus 100 mM phosphate is above the signal to noise ratio (S/N) of the Monolith NT.115 machine and binding can be concluded. Error bars indicate the standard deviation from the mean of four independent experiments. When a full titration was performed, protein was not saturated at concentrations $>$ 100 mM phosphate.
